# Cellular Events of Multinucleated Giant Cells Formation During the Encystation of *Entamoeba invadens*

**DOI:** 10.3389/fcimb.2018.00262

**Published:** 2018-07-31

**Authors:** Deepak Krishnan, Sudip K. Ghosh

**Affiliations:** Department of Biotechnology, Indian Institute of Technology Kharagpur, Kharagpur, India

**Keywords:** *Entamoeba*, haploidization, cell fusion, cytofission, nuclear fusion

## Abstract

*Entamoeba histolytica*, the causative agent of amoebiasis, does not form cysts *in vitro*, so reptilian pathogen *Entamoeba invadens* is used as an *Entamoeba* encystation model. During the *in vitro* encystation of *E. invadens*, a few multinucleated giant cells (MGC) were also appeared in the culture along with cysts. Like the cyst, these MGC's were also formed in the multicellular aggregates found in the encystation culture. Time-lapse live cell imaging revealed that MGC's were the result of repeated cellular fusion with fusion-competent trophozoites as a starting point. The early MGC were non-adherent, and they moved slowly and randomly in the media, but under confinement, MGC became highly motile and directionally persistent. The increased motility resulted in rapid cytoplasmic fissions, which indicated the possibility of continuous cell fusion and division taking place inside the compact multicellular aggregates. Following cell fusion, each nucleus obtained from the fusion-competent trophozoites gave rise to four nuclei with half genomic content. All the haploid nuclei in MGC later aggregated and fused to form a polyploid nucleus. These observations have important implications on *Entamoeba* biology as they point toward the possibility of *E. invadens* undergoing sexual or parasexual reproduction.

## Introduction

The *Entamoeba* species, like most parasitic microbes, is considered to be asexual as the mechanism enabled the parasites to generate clonal populations that have well adapted to host. Sexual or non-meiotic parasexual recombination could disrupt the allelic combinations required for the success of the parasite, and thus a clonal population structure was proposed initially for parasitic protozoa (Tibayrenc et al., 1990). Harmful mutations are supposed to accumulate in asexually reproducing organisms, and the sexual pathway is necessary to prevent this process known as Muller's ratchet. A clonal population from asexual reproduction reduces the genetic variability in the population required for adaptation and evolution. So the present understanding of sexual nature of parasitic protozoa is that they produce a clonal population by asexual pathway but retains sexual reproduction (Heitman, 2006). When met with environmental stress like antimicrobial therapy or host immune reaction sexual pathway produces a diverse progeny from which a new favorable trait can be selected and get fixed in the population through subsequent clonal lineages. Such strategy was reported in fungal pathogens where they show a clonal population structure but have evolved various sexual or parasexual mechanisms, and even rare sexual events were observed to change their pathogenicity and lifestyle (Ene and Bennett, 2014). Alteration of pathogenicity by sex was observed in *Toxoplasma* with the sexual process giving rise to hyper-virulent strains from avirulent parents (Grigg et al., 2001). The reason behind Vancouver Island *Cryptococcus gattii* outbreak was found to be a highly virulent strain produced by a cryptic unisexual mating (Fraser et al., 2005). These observations show that even rare events of sex could alter the lifestyle of a parasite and create public health problems. Thus, the understanding of the sexual pathway is of enormous medical importance especially in vaccine and drug development.

Observation of sexual or parasexual reproduction is difficult in most protozoan parasites as it was infrequent or occurred under unknown conditions, or it was not recognized as a sexual mechanism (Birky, 2005). But lately such mechanisms have been reported in important protozoan parasites like *Giardia* (Poxleitner et al., 2008), *Leishmania* (Akopyants et al., 2009), and *Trypanosoma* (Peacock et al., 2014). Generation of hybrids, detection of meiotic genes and population genetics (Weedall and Hall, 2015) have been used so far to find the presence of sexual reproduction in protozoa. Genome data analysis showed that *Entamoeba histolytica* and its reptilian counterpart *Entamoeba invadens* have most of the meiotic genes required for sexual/parasexual reproduction (Ramesh et al., 2005; Ehrenkaufer et al., 2013). Gene conversion by homologous recombination in Gal/GalNac lectin genes, which could help the parasite in immune evasion, has also been reported in *E. histolytica* (Weedall et al., 2011). Isolated parasites from the intestine and liver abscess of the same patient showed genetic variation indicating the presence of genomic reorganization and formation of parasites with invasive characteristics (Ali et al., 2008). Also, multi-locus sequence typing of *E. histolytica* isolates from the same geographic origin showed very high genomic diversity indicating DNA recombination (Gilchrist et al., 2012). All these observations indicate *Entamoeba* undergoes sexual or parasexual reproduction at some stage but how and when it takes place is not yet understood. Meiotic genes were found to be up-regulated (Ehrenkaufer et al., 2013) and homologous recombination was observed to be enhanced (Singh et al., 2013) during the stage conversion of reptilian parasite *Entamoeba invadens* which is used as a model for studying encystation process as *E. histolytica* does not form a cyst *in vitro*. Starvation triggered the sexual pathway in many eukaryotes like yeast and *Dictyostelium*, and *E. invadens in vitro* encystation is also a response to starvation. Cell fusion, nuclear fusion, and ploidy transitions are the hallmarks of sexual or parasexual events and so using microscopy, the encystation process of *E. invadens* was investigated for the presence of these events.

## Materials and methods

### Cells and reagents

*Entamoeba invadens* strain IP-1 was maintained in TYI-S-33 medium containing 10% adult bovine serum (HiMedia) and 3% Diamond vitamin mix at 25°C. DAPI, Propidium iodide, Hoechst 33342, Fluorescein diacetate, and calcofluor white were purchased from Sigma-Aldrich. Alexafluor 488 conjugated phalloidin was purchased from Molecular Probes, Invitrogen, USA.

### Encystation

To prepare the encystation induction (LG 47) medium which contained 47 % of nutrients, TYI medium without glucose was prepared and diluted to 2.12 times and completed with 5% heat inactivated adult bovine serum, 1.5% vitamin mix and antibiotics, penicillin and streptomycin. Mid log phase trophozoites were chilled on ice for 10 min to detach the cells from the culture tube wall and harvested by centrifugation at 500 × g for 5 min at 4°C. The cells were washed multiple times with LG media and 5 × 10^5^ trophozoites per ml were counted and transferred into encystation induction medium (LG) and incubated at 25°C. These cultures were observed for the presence of giant cells and used for further experiments.

### Cell staining

Cells were fixed with 4% (w/v) paraformaldehyde in PBS for 10 min and then permeabilized in 0.1% (v/v) Triton X-100 in PBS for 5 min. DAPI and PI were used to stain the nucleus. Chitin wall was stained with calcofluor white (Arroyo-Begovich et al., 1980). For actin localization permeabilized cells were blocked with 2% (w/v) BSA and stained with Alexafluor 488 conjugated phalloidin.

### Microscopy

Olympus FV1000 confocal microscope and Olympus IX 51 fluorescence microscope with camera attachment and photo-editing software (Image Pro Discovery) were used for imaging. From the images, cell cross sectional area and number of nuclei were measured using ImageJ software (NIH).

### Cell viability determination

Cells from encystation culture were pelleted and resuspended in PBS containing 10 μg/ml Fluorescein diacetate. After incubating at room temperature for 5 min cells were washed with PBS. The cells were then observed under a fluorescence microscope for the fluorescence produced by live cells.

### Live cell imaging

Live cell imaging was done using Olympus 1X51 inverted light microscope. Hoechst 33342 was used to stain nuclei for live cell imaging. Because of the high cell density and cell aggregates of encystation culture which obscured the live cell imaging, the cell aggregates were dispersed by adding galactose and the culture was diluted and then taken in tissue culture plates for imaging. The raw images were then processed using ImageJ software (http://rsb.info.nih.gov/ij/).

### Analysis of cell motility

Time-lapse video microscopy was performed using Olympus 1X 51 inverted microscope with camera attachment and photo-editing software (Image Pro Discovery). From the time-lapse recording, the trajectories of the cells were visualized using the ImageJ software (National Institutes of Health, Bethesda, USA) with plugins Manual Tracking and MTrack J. The tracking data was then analyzed using Chemotaxis and Migration Tool (ibidi) to obtain velocity and directness.

### Measurement of nuclear DNA content

Encystation cultures containing MGC and cyst were chilled to detach the cells, pelleted by centrifugation at 500 g, washed with ice-cold PBS, and then fixed in 4% (w/v) paraformaldehyde in PBS for 10 min. It was then permeabilized in 0.2% (v/v) Triton X-100 in PBS for 5 min and blocked with 2% (w/v) BSA in PBS. After treating with 20 μg/ml RNase A, the nuclei were stained with 10 μg/ml Propidium Iodide. Olympus FV1000 confocal microscope was then used to take image of the cell. From this image fluorescence intensity of trophozoite and MGC nuclei were measured using ImageJ (NIH). The results were represented using Box whisker plot. Boxes indicate 25–75 percentiles; the line 50th percentile and the solid square indicate the mean. The whiskers mark the 10th and 90th percentile.

### Statistical analysis

Quantitative data are shown as the mean ± standard deviation (SD). The significance of the experimental data was calculated using unpaired, two-tailed Student's *t*-test. Differences between groups were considered statistically significant if *p* < 0.05.

## Results

### Multinucleated giant cells are formed during encystation by cell fusion

**Figure 1:**
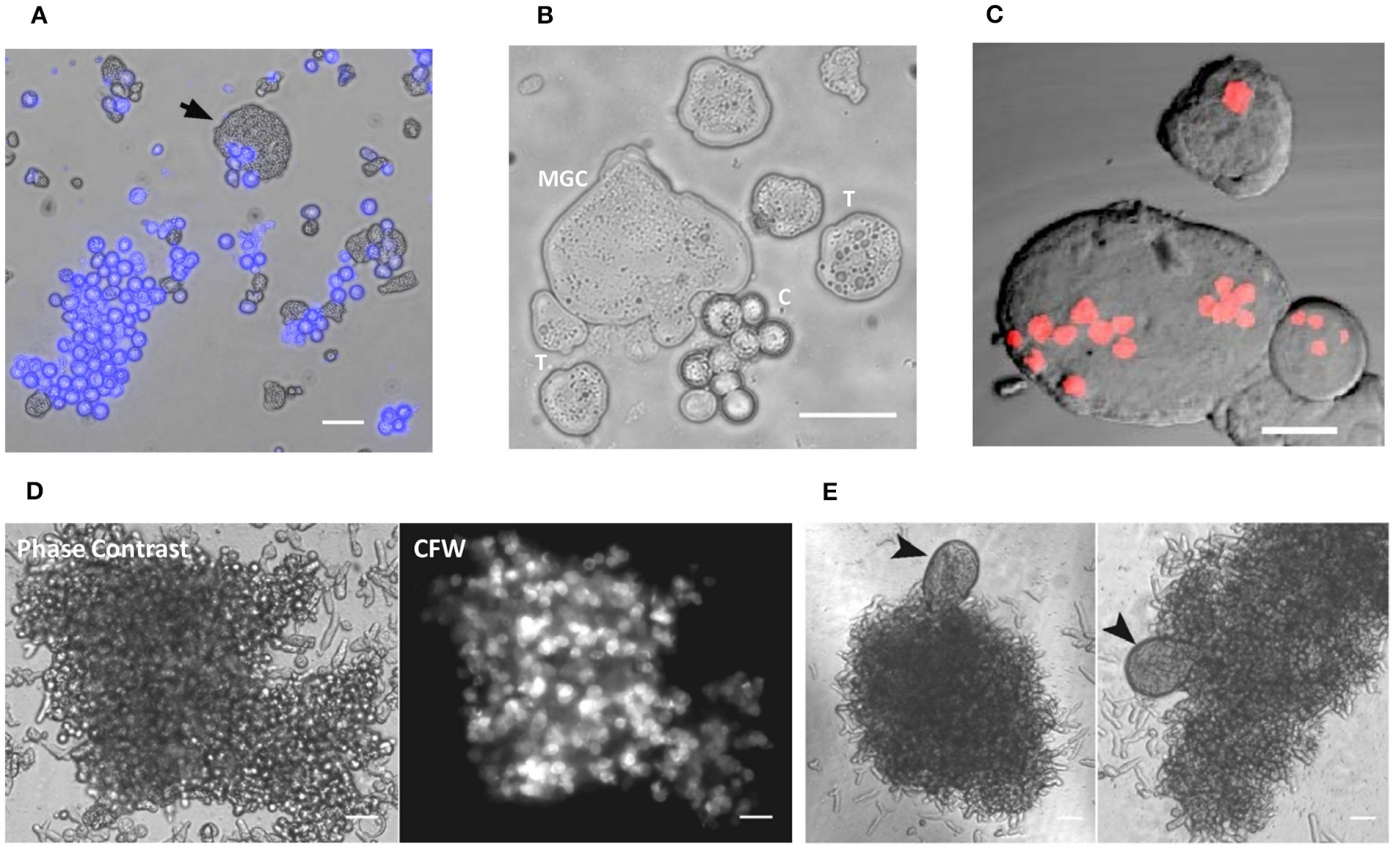
Multinucleated giant cells formed during encystation. **(A)** Encystation culture sample showing cyst (Calcofluor white stained) and the giant cell (arrow mark) **(B)** Trophozoites, cysts and giant cells taken from encystation culture (T: trophozoites, C: cysts). See also Movies S1. **(C)** Nuclear staining with PI showing the multinucleated nature of MGC (Scale bars: 10 μm). **(D)** Cell aggregates with the cysts stained with calcofluor white found in the encystation culture. **(E)** MGC's were observed associated with these cell aggregates. Scale bars: 50 μm.

**Figure 2:**
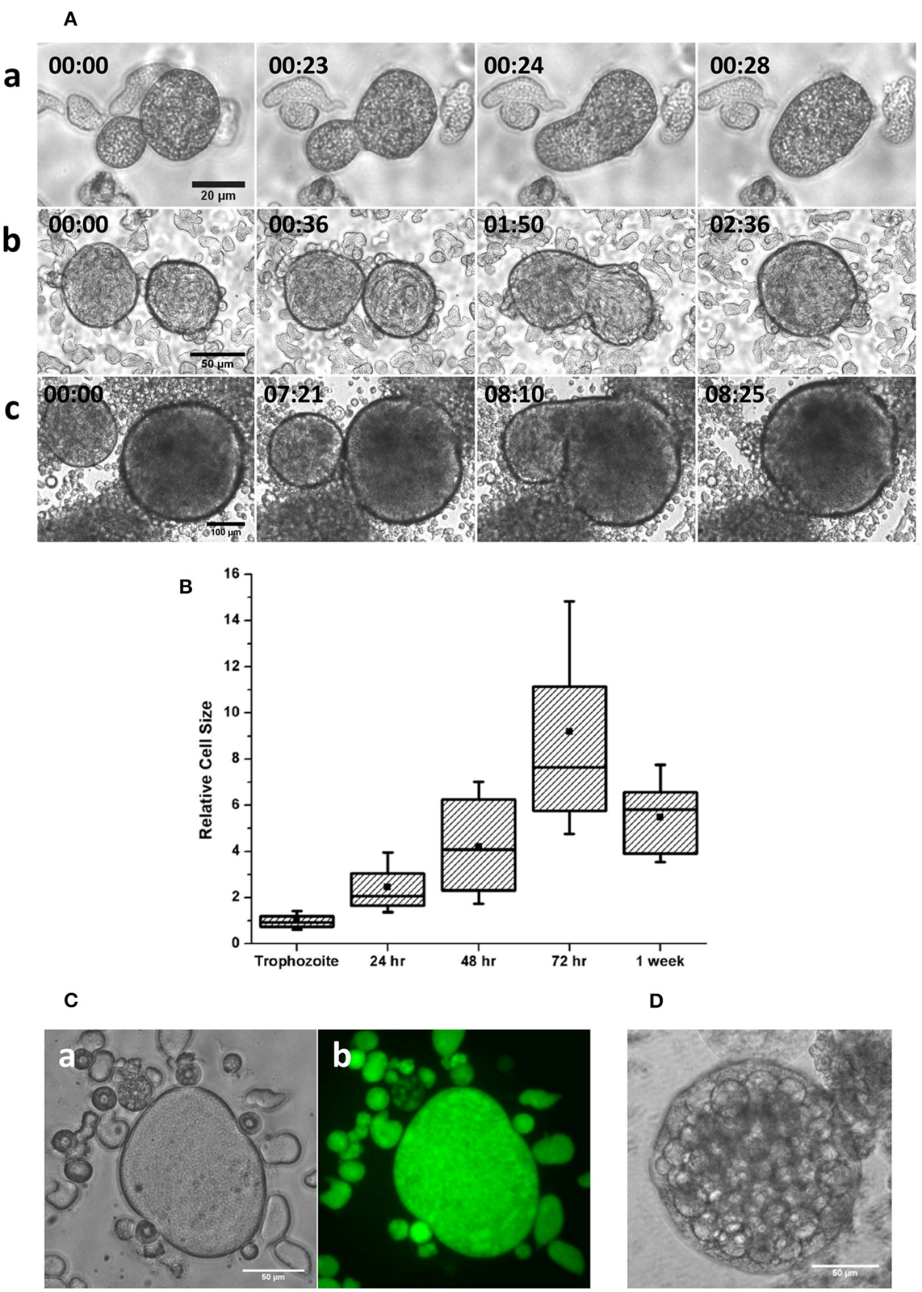
Cell fusion in multinucleated giant cells **(A)** Stages of cell fusion in MGC obtained from 36 **(a)**, 48 **(b)**, and 72 h **(c)** (Scale bars: **a**: 20 μm; **b**: 50 μm; **c**: 100 μm). See also Movie S2. **(B)** The size distribution of MGC obtained from encystation cultures at different intervals relative to trophozoites shows doubling of size in the early hours. **(C)** Fluorescein diacetate (FDA) hydrolysis assay showed that the MGC from older culture found inside aggregates **(a)** produced and retained Fluorescein **(b)** indicating their viability. **(D)** Very large MGC formed by continuous fusion in the media sometimes underwent vacuolation and cell lysis. Scale bars: 50 μm.

Encystation model organism *Entamoeba invadens* undergoes stage conversion to produce chitin walled cyst when subjected to starvation and osmotic stress (Sanchez et al., 1994). It was observed that during encystation, a few giant cells also appeared in the culture (Figures 1A,B and Movie S1) along with cyst, and nuclear staining showed these cells were multinucleated (Figure 1C). The MGC were observed at a very low frequency (1 in 10^4^ cells), and size of observed giant cells showed considerable variation. *Entamoeba invadens* cysts were formed only inside the cell aggregates found in the encystation culture (Figure 1D) and MGC were usually observed emerging from or associated with these cell aggregates (Figure 1E). The cell aggregates were galactose ligand-mediated (Cho and Eichinger, 1998) and the addition of galactose to the LG medium disrupted of the cell aggregates and released these multinucleated giant cells (MGCs) into the medium. To study their behavior released MGC were observed using time-lapse live cell imaging. The MGC obtained from early encystation cultures (24–48 h) did not show any cell adhesion and moved randomly in the media. When these early MGC came into contact with each other, their membrane fused very rapidly at the point of contact, and they became one cell. When kept together in the media MGC fused continuously to produce bigger and bigger cells (Figure 2A and Movie S2). The cell fusion continued even after transferring the MGC to growth media (TYI-S-33). The cell fusion was very fast in early MGC (24–36 h) with the cells fusing instantaneously on membrane contact. Larger MGC from 48 to 72 h of encystation took longer time, and MGC obtained after 96-h culture did not fuse. To find how the cell size changes with time, MGCs were obtained from encystation cultures at different time points and their cross-sectional area, which is an approximate measure of cell size, was compared to that of trophozoites (Figure 2B). MGC became observable by 24th hour, and these early giant cells were nearly twice the size of trophozoites, and by 48th hour their size again increased two-fold by fusing among themselves. MGC obtained from 72 to 96 h showed a broad distribution in size with a few of them reaching sizes 10 times to that of trophozoites, but MGC from week old cultures were comparatively smaller. The doubling of cell size in the early hours and the ability to undergo continuous fusion implies that the starting point of MGC could be the trophozoites which acquired fusion competency. The MGC's were never observed to interact or fuse with trophozoites indicating cell surfaces changes like the expression of fusion proteins. Cell signaling required cyst formation take place inside the cell aggregates (Coppi et al., 2002) and as MGC were often seen associated with these aggregates, the formation of fusion- competent trophozoites and the initial cell fusions may also be occurring inside them. *Entamoeba* genome was searched for all known proteins involved in cell fusion, but none could be detected yet. Fluorescein diacetate (FDA) hydrolysis assays showed that most MGC, especially those remained inside cell aggregates retained their viability even in week-old cultures (Figure 2C) though a few MGC released into the media sometimes underwent vacuolation and cell lysis (Figure 2D). Multinucleated cells are regularly found in *Entamoeba histolytica* culture (Supplementary Figure 1), but time-lapse live-cell imaging did not show the presence of cell fusion. These cells were probably formed due to the delinking of nuclear division and cytokinesis as cell cycle checkpoints are absent in *Entamoeba histolytica* (Das and Lohia, 2002).

### Multinucleated giant cells undergo cytofission in confinement

**Figure 3:**
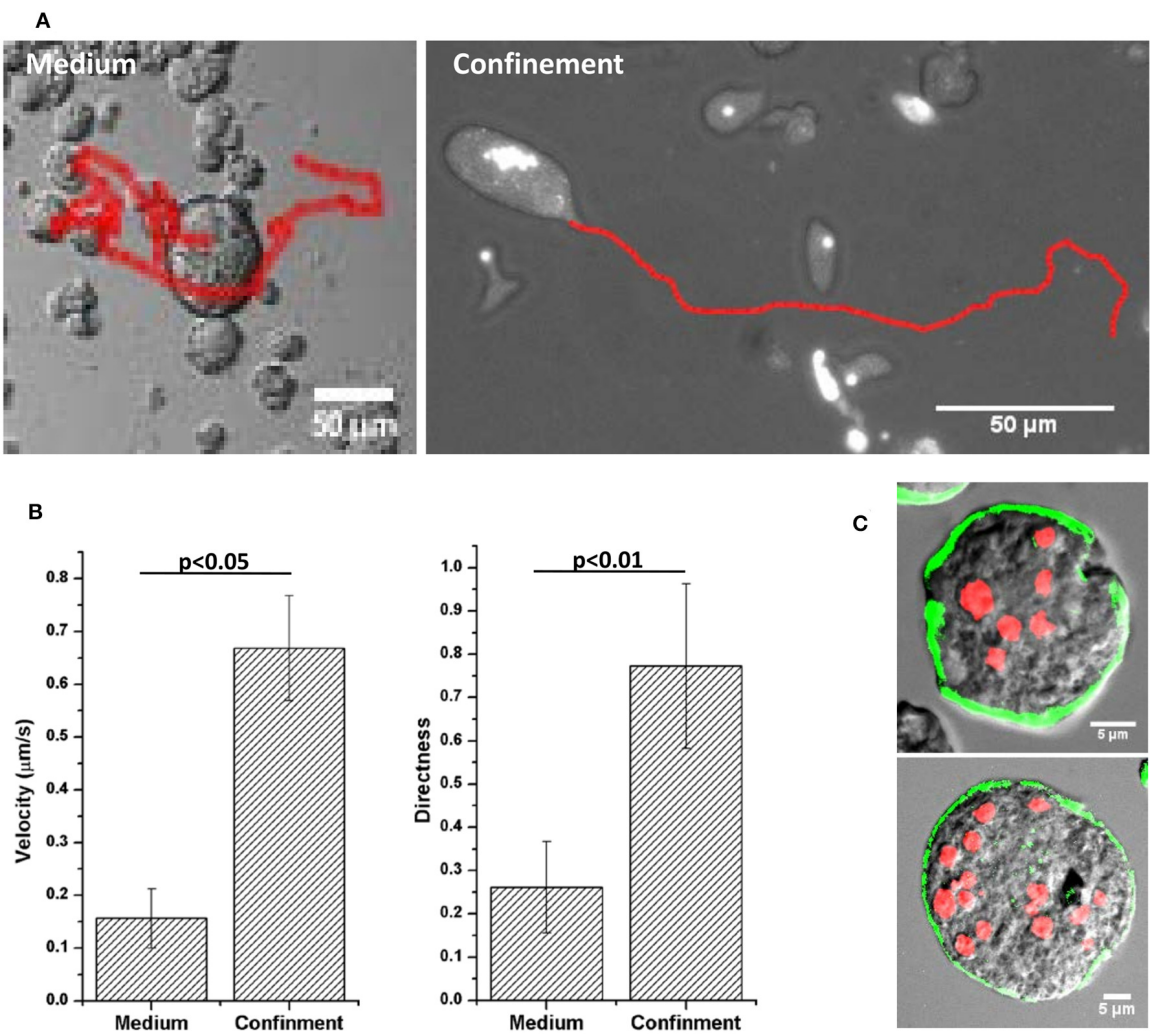
Motility of MGC in medium and under confinement. **(A)** Migration tracks of MGC in the medium and under confinement. **(B)** Velocity and Directness (ratio of distance between the start and the end point and the total distance traversed by the cell) of the MGC motility. Velocity in the medium was 0.15 ± 0.05 μm/s and under confinement it increased to 0.66 ± 0.09 μm/s. The MGC moved randomly in the media as shown by the directness value 0.26 ± 0.10 and under confinement the migration became directionally persistent with the directness value of 0.77 ± 0.19. **(C)** Alexafluor 488-phalloidin staining of early motile MGC showed actin was present in the cortical region of the MGC. Scale bars: 5 μm.

The MGC released from the aggregates moved very slowly in the culture media (Movie S3) but when compressed between slide and cover slip the MGC became highly motile (Figure 3A). To analyze the changes in cell motility, its velocity and directness was measured (Zengel et al., 2011). Directness is the ratio of the displacement and the total distance traversed by the cell and is a measure of directional persistence (lower values indicting random motility and values closer to one indicating directional motility). Velocity of the cell in the liquid media was found to be 0.15 ± 0.05 μm/s, and the directness value of 0.26 ± 0.10 indicting random motility (Figure 3B). In confinement velocity of the MGC increased to 0.66 ± 0.09 μm/s and the motility became highly directional as shown by the directness value of 0.77 ± 0.19. Staining the early MGC with Alexafluor 488- phalloidin showed no cell adhesion structures but the presence of actin under the plasma membrane (Figure 3C). In the absence of cell–substrate adhesions, early MGC could be using chimneying motility, in which the cell generate traction using the opposing surfaces to crawl in a 3D environment and that required the actomyosin cortex (Malawista et al., 2000; Hawkins et al., 2009; Liu et al., 2015). The cell aggregates in the encystation culture could be providing the confinement as MGC were associated with them.

**Figure 4:**
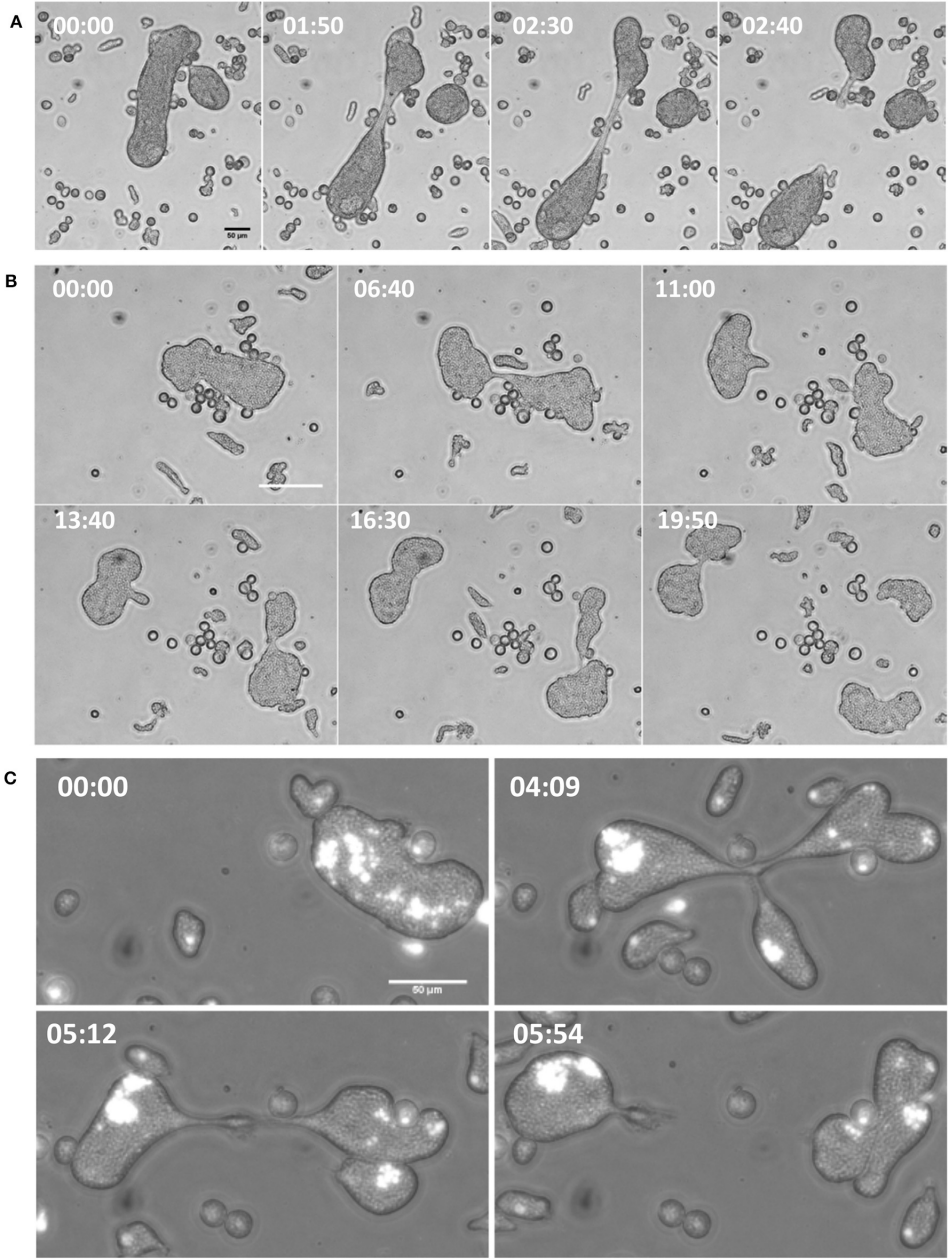
Increase in velocity under confinement led to cytofission in MGC. **(A)** MGC underwent cytoplasmic fission in confinement by moving in opposite direction and tearing the cell in to two.**(B)** The daughter cells were also capable of continuous cytofission. See also Movies S4–S6 **(C)** Random nuclear distribution during cytofission process shown by Hoechst 33348 staining. (Scale bars: 50 μm, time in minutes). See also Movie S7.

**Figure 5:**
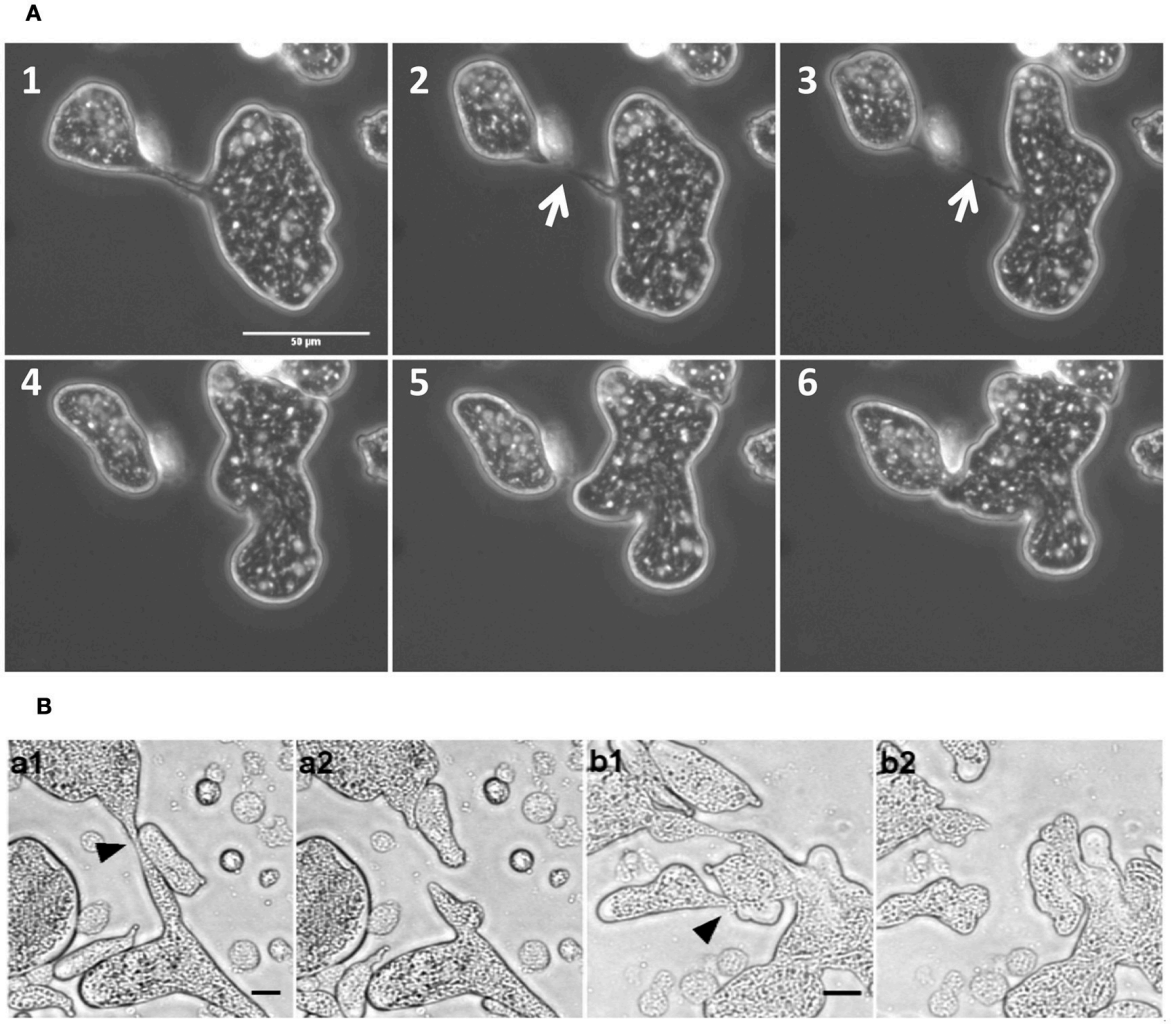
MGC cytofission was assisted by trophozoites. **(A)** When the membrane bridge linking the dividing cells (arrow mark) was not severed, the daughter cells came back and re-joined. Scale bars: 50 μm **(B)** Trophozoites converged on the dividing area **(a1,b1)** and severed membrane bridge between two daughter cells **(a2,b2)**. See also Movies S8 and S9. Scale bars: 20 μm.

The consequence of such increased motility was the rapid cytoplasmic fission of MGC due to movement toward opposite directions and tearing itself into daughter cells (Figure 4A and Movie S4). The resulting daughter cells were also capable of further cytofission (Figure 4B and Movie S5), and these divisions often continued until all the daughter cells reached trophozoite size (Movie S6). When the distribution of nuclei into daughter cells during cytofission was observed using cell-permeable nuclear stain Hoechst 33342, it was found that there is no correlation between daughter cell size and the number of nuclei per cell (Figure 4C and Movie S7). Cytofission started at random sites, and nuclei were unevenly distributed, so that daughter cells with variable numbers of nuclei were formed. The daughter cells of cytofission sometimes remained connected through the cytoplasmic bridge (Figure 5A). It was observed that the nearby trophozoites converged on the dividing area probably helping the cytofission by severing cytoplasmic bridge between two daughter cells (Figure 5B and Movie S8, S9) as reported to occur during *E. invadens* cell division (Biron et al., 2001).

**Figure 6:**
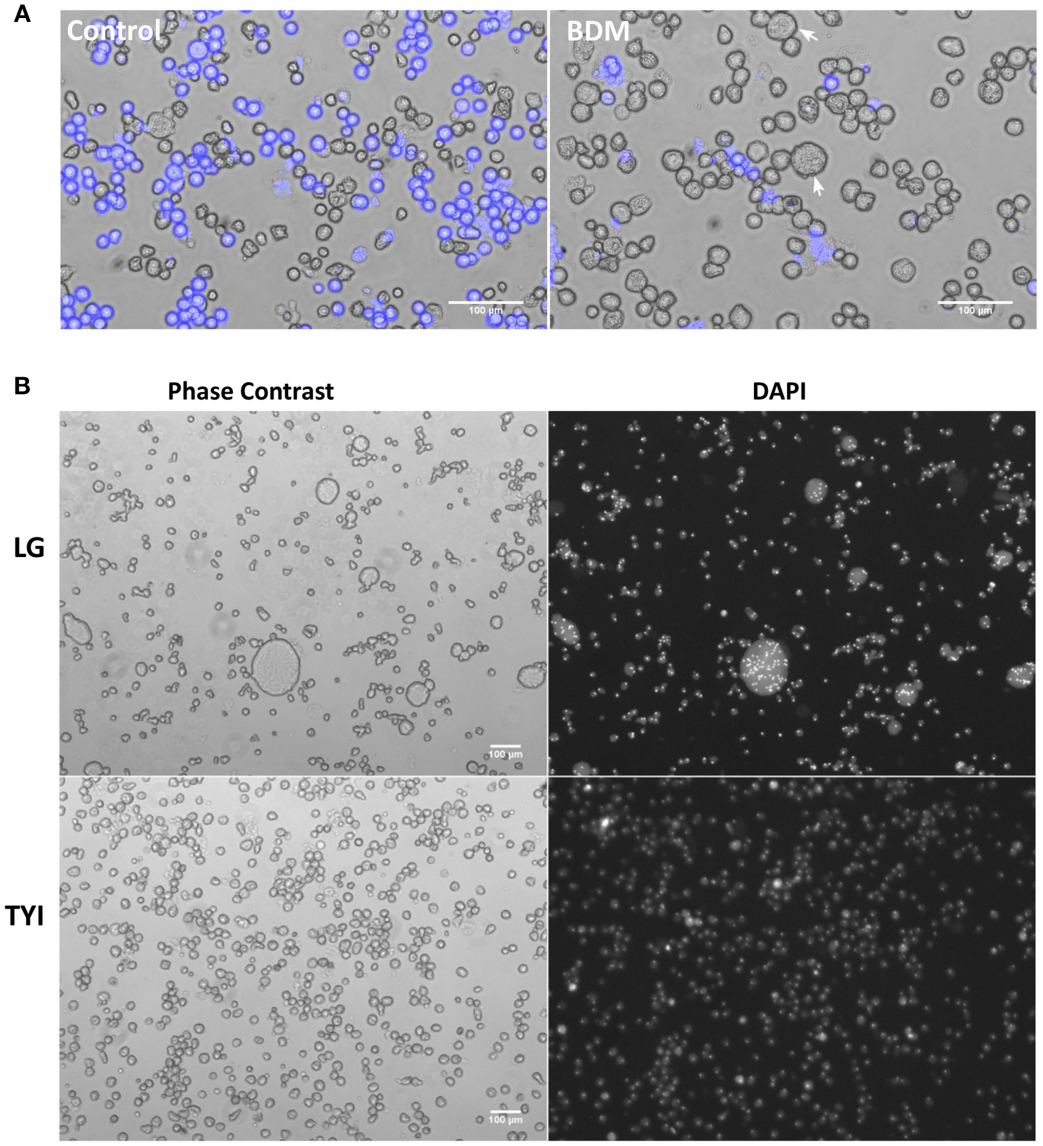
Effect of Myosin II inhibitor 2, 3-butanedione monoxime (BDM) on MGC formation. **(A)** Addition of 20 mM BDM inhibited encystation as shown by CFW staining but caused the formation of giant cells (arrow mark). **(B)** 48th hour BDM treated encystation culture (LG) stained with DAPI showing the increase in the number of MGC. Addition of BDM to growth medium (TYI) did not cause multinucleation. Scale bars: 100 μm.

Myosin II inhibitor, 2,3-butanedione monoxime (BDM) was observed to inhibit the encystation when added to the encystation culture at a concentration 20 mM (Figure 6A) but it greatly increased the number of MGC to 1 in 10^2^ (Figure 6B). In growth media, BDM treatment did not cause any multinucleation, so the development of giant cells was not due to cytokinesis failure and it also did not inhibit the cell aggregation step during encystation. As actomyosin contractile ring is responsible for cleaving the dividing cell into two daughter cells, BDM might have blocked the cytofission without stopping the cell fusion, thus the number of MGC was found to be increased. These observations indicate the possibility of continuous fusion and cytofission occurring inside the cell aggregates and the MGC's observed in older encystation cultures could be the final product of such cyclic fusion and cytofission. The galactose ligand mediated cell aggregate is required only in the early hours of encystation in encystation medium (LG) to provide the cell signaling (Turner and Eichinger, 2007). During the *in vitro* encystation most of the cells in the multicellular aggregates were converted to cysts. Once the cysts were fully formed, aggregates lost their compact structure and release the cyst and MGC, if there is any into the media.

### MGC nuclei undergo haploidization after cell fusion

**Figure 7:**
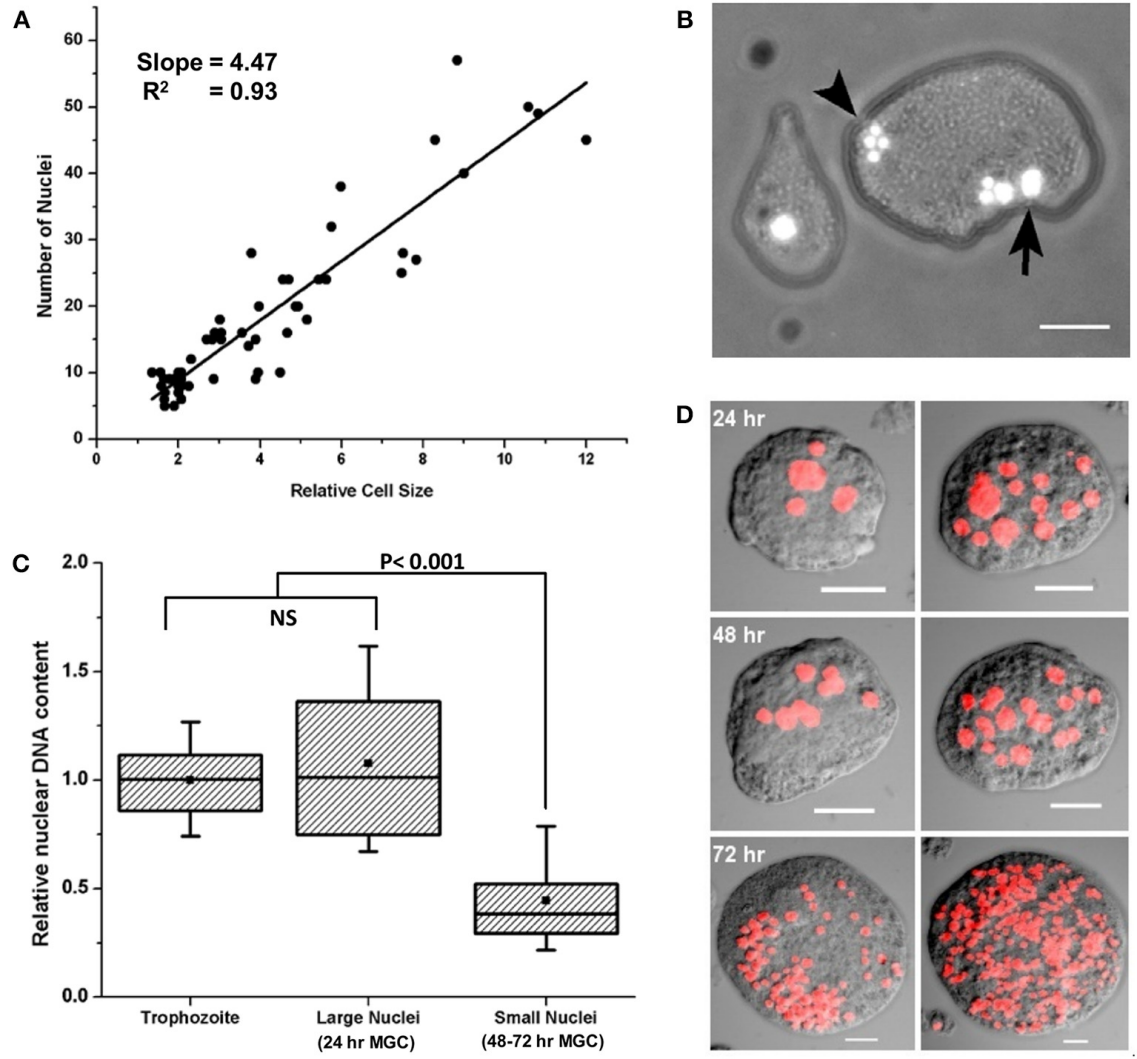
MGC nuclei undergo division after cell fusion. **(A)** Scatter plot of relative MGC size vs. number of nuclei (*N* = 77) showed that the relationship could be explained as Number of nuclei per MGC ≈ 4 × increase in MGC size relative to trophozoite. Thus for each fusion competent trophozoite participated in fusion, number of MGC nuclei increased fourfold (Slope = 4.47, *R*^2^ = 0.93). **(B)** Early MGC contained nuclei of different sizes (See also Movie S10). **(C)** Comparison of DNA content of trophozoite nuclei (*N* = 355), large nuclei found in 24 h MGC (*N* = 36) and small nuclei found in 48–72 h MGC (*N* = 563). Nuclear content of large nucleus was similar to trophozoite nucleus but the smaller nuclei were haploid relative to trophozoites. Boxes indicate 25–75 percentiles; the line 50th percentile and the solid square indicate the mean. The whiskers mark the 10th and 90th percentile (NS: Not significant). **(D)** Representative images of MGC from different hours of encystation showing the changes in number and size of nuclei after staining with PI. MGC from 24 h of encystation contained larger nuclei along with haploid nuclei (Upper). MGC took from 48 h of encystation show mainly haploid nuclei (Middle). MGC from 48 to 72 h of encystation were formed by the fusion of smaller MGC (Lower). These cells also contained only haploid nuclei (See also Movie S11). Scale bars: 10 μm.

To find the changes in MGC nuclei following cell fusion, their number, size and genomic content were measured and compared with that of trophozoites. Since each MGC were formed by the continuous fusion of the trophozoites which gained fusion competency, the number of nuclei must be proportional to cell size. When the increase in MGC size relative to trophozoite estimated from the cross-sectional area was plotted against the number of nuclei per cell (Figure 7A), their relationship could be explained as

Number of nuclei per MGC ≈ 4 × fold increase in MGC size (relative to size of trophozoites)

This meant that for each fusion competent trophozoite participated in the formation of MGC; there was a fourfold increase in the number of nuclei. The early MGC were usually found to contain nuclei of different sizes, large nuclei with size similar to that of trophozoites, and clusters of small nuclei (Figure 7B and Movie S10). To find the genomic content of these nuclei, MGC were isolated from encystation culture at different time points, their nuclei were stained with Propidium Iodide (PI), and images were taken using confocal microscope. From the image, the fluorescence intensity of each nucleus which is a measure of genomic DNA content was calculated. *E. invadens* trophozoites exhibited a broad distribution of DNA content within a population due to lack of cell cycle control (Byers and Eichinger, 2005; Mukherjee et al., 2009). The distribution of nuclear DNA content of the larger nuclei observed in 24th hour MGC was similar to that of trophozoites, but in smaller nuclei, it reduced by half (Figure 7C). The large nuclei were only observed in MGC from 24th hour and small nuclei were prevalent in MGC obtained from 48 to 72 h (Figure 7D), so nuclear division in MGC probably started after the initial cell fusions between fusion competent trophozoites.

### Nuclear aggregation and fusion occur in late MGC

**Figure 8:**
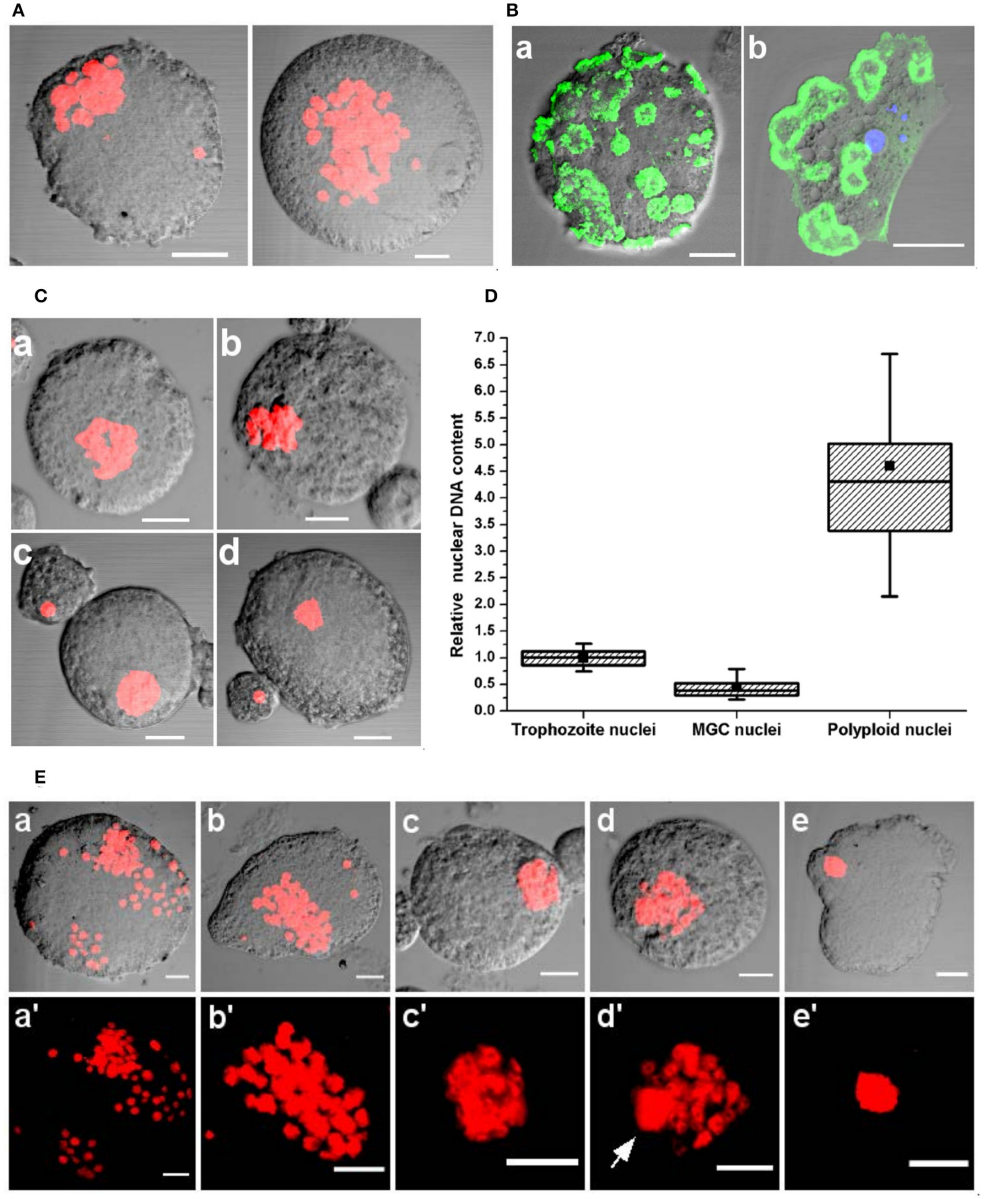
Nuclear aggregation and fusion in MGC. **(A)** PI staining shows nuclear aggregation observed in MGC taken from encystation cultures after 72–96 h of incubation. See also Movie S12. **(B)** Staining the non-motile MGC with Alexafluor 488 phalloidin showed the presence of actin structures on the cell surface **(a)** which could be podosome rosettes found on the ventral side of adherent *E. invadens* trophozoites **(b)**. **(C)** In week old cultures most MGC contained closely packed nuclei **(a,b)** or a single polyploid nucleus **(c,d)** were found in encystation cultures after week long incubation. **(D)** Comparison of the nuclear content of trophozoite (*N* = 355), MGC (*N* = 563) and the giant cell with single nucleus (*N* = 20). **(E)** Stages of nuclear interactions leading to nuclear fusion. After 72 h nuclei started aggregating **(a–b')** to form a compact nuclear cluster **(c,c')** in which formation of a single large nuclei (**d,d'**, arrow mark) was observed. The nuclei which did not participate in fusion then disappeared to produce a giant cell with a single large nucleus **(e,e')**. Scale bars: 10 μm.

MGC taken from encystation cultures after 72–96 h of incubation were non-motile, relatively smaller, and adherent and nuclear staining showed all the nuclei had aggregated to one point (Figure 8A and Movie S11). As MGC became non-motile, the cortical actin reorganized into ring-like structures on the cell surface (Figure 8Ba). These structures were similar to podosome rosettes involved in adhesion and composed of individual podosomes arranged in a ring like shape, similar to those found on the ventral side of adherent trophozoites (Figure 8Bb). In week old encystation contained highly condensed clusters of nuclei (Figures 8Ca,b) and a few cells contained a single large nucleus (Figures 8Cc,d) which was found to be polyploid on nuclear DNA measurement (Figure 8D). This shows the presence of interaction among nuclei in older MGC leading to nuclear fusion (Figure 8E). These interactions start with nuclei clustering to one location (Figures 8Ea,a′,b,b′) and later these aggregates became tightly packed structure with individual nuclei becoming indistinguishable (Figures 8Ec,c′). Inside these clusters, a single large nucleus was sometimes observed (Figures 8Ed,d′) possibly resulting from nuclear fusion but it is unclear how many nuclei participated in fusion to produce the giant cell with a polyploid nucleus (Figures 8Ee,e′).

## Discussion

Life cycle of *Entamoeba histolytica* and *Entamoeba invadens* contains two stages, uninucleate motile trophozoites, and tetranucleate dormant cysts. Microscopic analysis of the *in vitro* encystation culture of *E. invadens* revealed rare encystation specific multinucleated giant cells formed by cell fusion. Multinucleated cells were also found in the stationary phase of *E. histolytica* culture, but it is reported to be due to cytokinesis failure, not cell fusion (Das and Lohia, 2002). The starting point for MGC was the conversion of a few trophozoites into fusion competent cells which may have involved the expression of fusion proteins since MGC never fused with normal trophozoites. It has been already reported that the encystation is initiated by the cell signaling taking place inside the cellular aggregates (Coppi et al., 2002). Similarly signaling within the aggregates may play some role in the formation of MGC. The high population density of the cell aggregate may also be required to increase the chance of cell fusion (Woznica et al., 2017). The MGC released from cell aggregates moved slowly and randomly in the media, but under confined conditions, they became highly motile and directionally persistent. The increased motility led to larger MGC tearing itself into smaller cells by cytoplasmic fission. Inhibition the cytofission by BDM greatly increased the number of MGC in the encystation media indicating sequential fusion and cytofission may be taking place inside the cell aggregates. Such cyclic fusion and cytofission could be the reason for variation observed in the number and size of MGC during encystation experiments. Formation of multinucleated cells that underwent cell fusion and cytofission was also observed during early stages of macrocyst formation which is the sexual stage of social amoeba *Dictyostelium discoideum* (Ishida et al., 2005) and also during the parasexual stage of amoeba *Cochliopodium* (Tekle et al., 2014). The exact relevance of such a random cell fusion and cytofission process is unclear, but it could help to share nutrients among cells during starvation and also gather different traits from multiple cells so that multinucleated cell which accumulated the best combination could survive during stress.

During the encystation, uninucleate trophozoite undergoes nuclear division to form tetranucleate cyst (Supplementary Figure 2). Correlation between increase in MGC size relative to trophozoites and number of nuclei it contained showed that for each trophozoite participated in the cell fusion to form MGC there was a fourfold increase in the number of nuclei indicating MGC also underwent similar nuclear division. But the final ploidy was different as the cyst nucleus contained one-fourth DNA (Lohia, 2003; Supplementary Figure 2) and MGC nuclei contained half genomic DNA compared to trophozoites. Ploidy changes can occur through meiotic or non-meiotic mechanisms like chromosome loss (Bennett and Johnson, 2003; Bennett et al., 2014). Meiotic genes were also reported to be expressed during encystation (Ehrenkaufer et al., 2013); but it is not yet clear whether the nuclear division in MGC is meiotic or not. Expression of meiotic genes also took place during the parasexual reproduction of *Giardia* (Carpenter et al., 2012) and *Candida albicans* (Forche et al., 2008). So it is important to determine the exact nature of ploidy transition in MGC. Nonetheless, fusion competency, fast motility, and presence of haploid nuclei make MGC the gamete equivalent of *Entamoeba*. In the late, non-motile MGC, the nuclei aggregated and underwent multiple nuclear fusions to form a polyploid nucleus. Both *E. invadens* and *E. histolytica* have been shown to alter ploidy depending on the growth conditions and different stages of life cycles (Mukherjee et al., 2008). It is yet to be determined whether the polyploid giant cells are returning to the trophozoite stage with respect to cell size and DNA content with time or not. Cell fusion or fertilization is the most important step in the sexual/parasexual pathways as it allows genetic exchange within the population and recombines beneficial traits from different lineages. The observation of cell fusion at any time during life cycle alone provides indirect evidence for the presence of sexual/parasexual pathway (Lahr et al., 2011). Even without any genomic recombination such cell fusion events (agamic cell fusion) can be important because through the increased nutrient reserves and resulting polyploidy and hybrid vigor, it can increase cell survival in adverse conditions (Comai, 2005; Goodenough and Heitman, 2014). The formation of multinucleated and polyploid cells by cell fusion was also reported in many cancers, and the resulting genome reorganizations facilitated metastasis and drug resistance in cancer (Weihua et al., 2011; Niu et al., 2016). Similarly, the MGC also possesses the potential to induce phenotypic variations in *Entamoeba* though this hypothesis is yet to be tested.

The three main developmental pathways of amoebozoans: sporulation, encystation and sexual macrocyst pathway were controlled by the environmental conditions (O'Day and Keszei, 2012). It could be possible that like encystation, MGC pathway is a stress response mechanism and activated by the nutrient and osmotic stress caused by the encystation media. Sexual/parasexual recombination causes mixing and reshuffling of genes and creates genetic variations in the population, but such events may be detrimental to a parasite as it disrupts the allelic combinations required for survival in the host. Most parasites have thus limited the sexual/parasexual reproduction to during stress or dispersal to new hosts (Weedall and Hall, 2015). While encystation helps to survive adverse conditions by forming a resistant cyst, MGC pathway can introduce hybrid fitness and beneficial genomic changes and ensure the survival of *Entamoeba* in a changing environment. But so far no other stress conditions tested like oxidative or heat shock, induced MGC formation. The *in vitro* encystation is conducted using an axenic culture but the intestinal bacteria have been shown to influence characteristics like DNA content and virulence (Bracha and Mirelman, 1984; Mukherjee et al., 2008) and it may also have an influence on the sexual/parasexual pathway. For example in the Choanoflagellate *Salpingoeca rosetta*, sexual reproduction is enhanced by bacteria *Vibrio fischeri* by inducing cell aggregates in which the cells underwent extensive fusion (Woznica et al., 2017).

Like cysts, MGC were also formed inside cell aggregates, so the initial cell signaling associated with encystation or the expression of meiotic genes may have caused a few cells to gain fusion competency and start the MGC pathway. Encystation is the ancestral survival mechanism of all amoebozoa (Kawabe et al., 2009) and the cysts themselves can be asexual or sexual where a zygote formed by cell fusion undergoes encystation (Schaap and Schilde, 2018). *Copromyxa protea* and *Sappinia diploidea* form double walled sexual cyst by fusion of two amoebae (Walochnik et al., 2010; Brown et al., 2011) but no such wall formation was observed in MGC. Sexual reproductions and encystations were probably present in the life cycle of last eukaryotic common ancestor (LECA). Cell fusion and meiosis during encystation might have helped them to survive as dormant cysts in adverse environmental conditions by providing genetic redundancy and recombinational DNA repair, and that may be associated with the evolution of sex (Cavalier-Smith, 2010). *Entamoeba* is regarded as a primitive eukaryote (Bakker-Grunwald and Wöstmann, 1993) and even if the MGC cell fusion is agamic, the study of its cellular events could be useful in understanding the origin and evolution of sexual reproduction.

## Author contributions

DK and SG: designed research; DK: performed research; DK and SG: wrote the paper.

### Conflict of interest statement

The authors declare that the research was conducted in the absence of any commercial or financial relationships that could be construed as a potential conflict of interest.
