# Supplementary material for: Cellular Events of Multinucleated Giant Cells Formation During the Encystation of Entamoeba invadens
Source: Front Cell Infect Microbiol. 2018 Jul 31;8:262. doi: 10.3389/fcimb.2018.00262 (PMC6079502; doi:10.3389/fcimb.2018.00262)
Supplement: Supplementary file 13 [file Data_Sheet_1.docx]

Supplementary Material

Evidence of sexual reproduction in *Entamoeba invadens* and its cellular events

Deepak Krishnan, Sudip Kumar Ghosh *

*** Correspondence:** Sudip Kumar Ghosh: [sudip@hijli.iitkgp.ernet.in](mailto:sudip@hijli.iitkgp.ernet.in)

## 1. Supplementary Figures


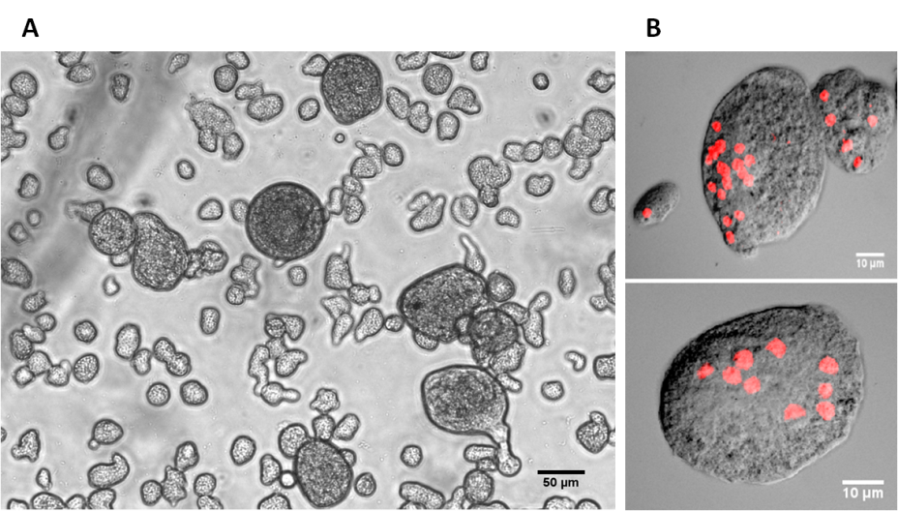


**Supplementary Figure 1.** **(A)** *Entamoeba histolytica* multinucleated giant cells. Scale bars: 50 µm **(B)** Multinucleated giant cells found in stationary phase culture stained with PI. Scale bars: 10 µm

**
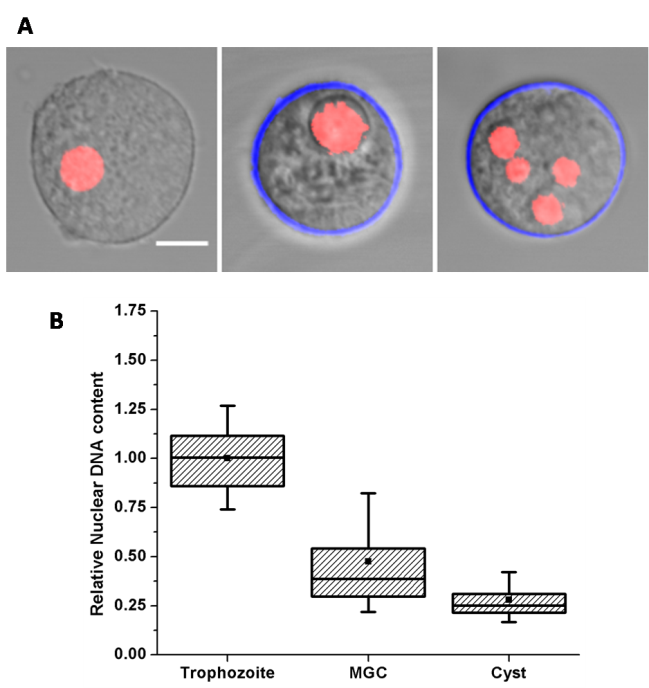
**

**Supplementary Figure 2. (A)** Staining the chitin wall (Calcofluor white) and nuclei (PI) shows the formation of tetranucleate cyst from trophozoite. Scale bars: 5 µm. **(B)** Comparison of trophozoite (N= 355), MGC (N= 576) and cyst (N= 86) nuclear DNA content. MGC nuclei contained half the genome and mature cyst nuclei contained one forth genome compared to trophozoites.

**2. Movies**


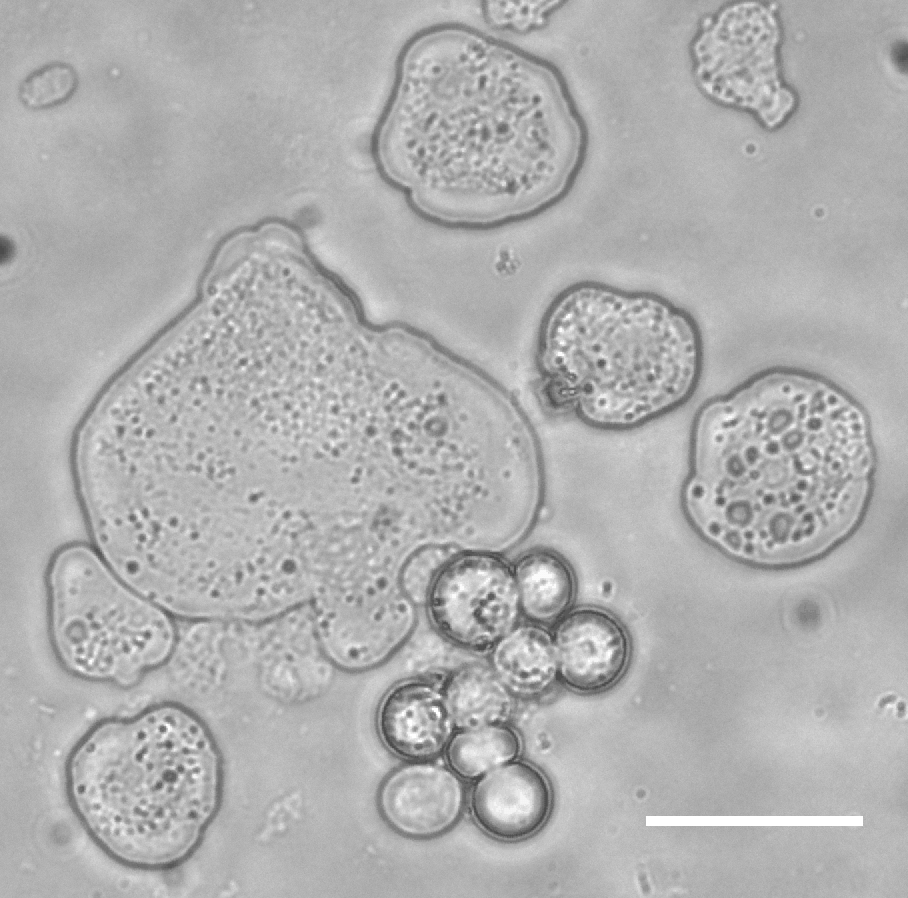


Movie S1. Trophozoites, cysts and MGC taken from encystation culture.


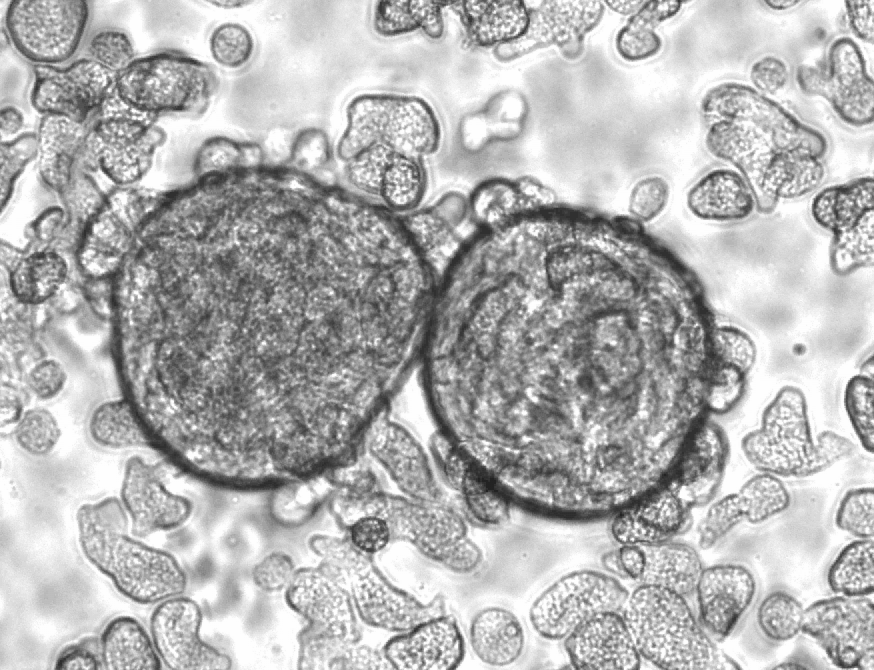


Movie S2. Cell fusion shown by MGC of different size.


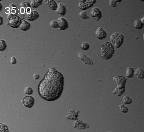


Movie S3. MGC motility in medium.


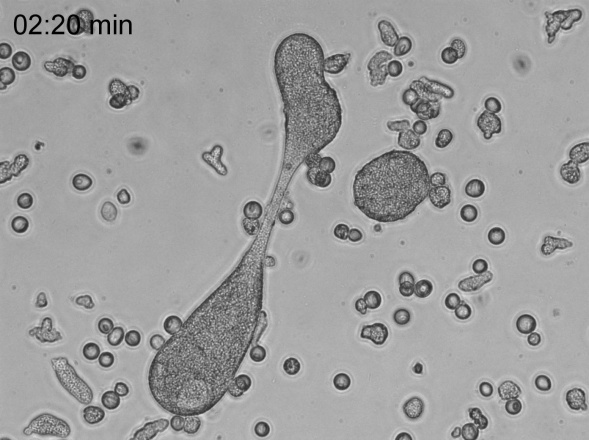


Movie S4. Cytofission shown by MGC. Trophozoites and cysts can also be seen.


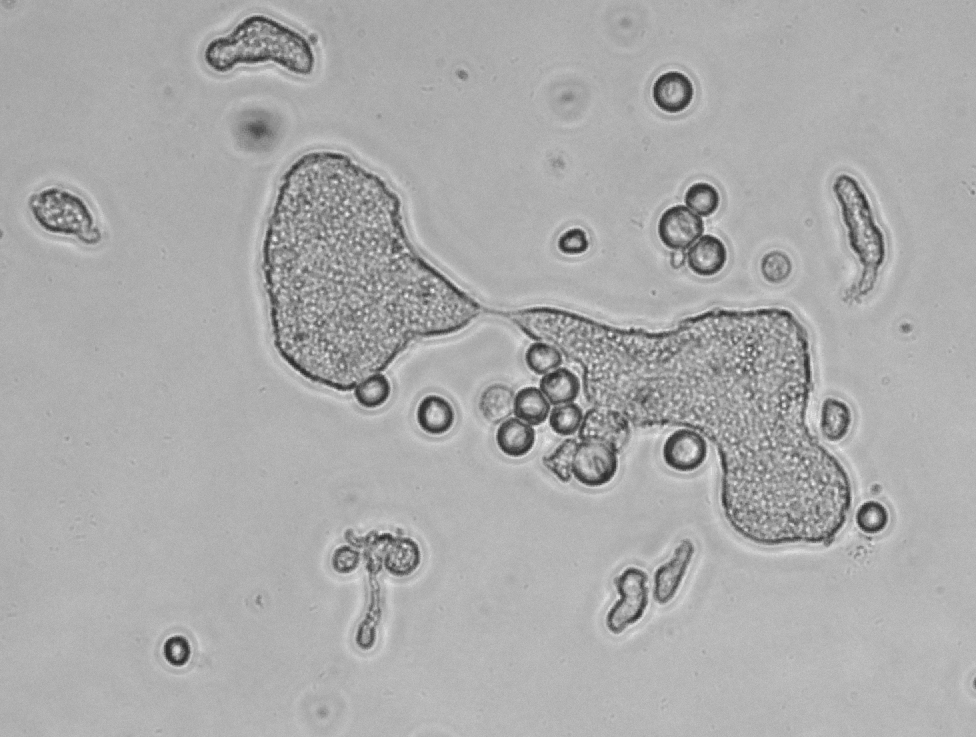


Movie S5. Sequential cytofission shown by MGC. Trophozoites and cysts can also be seen.


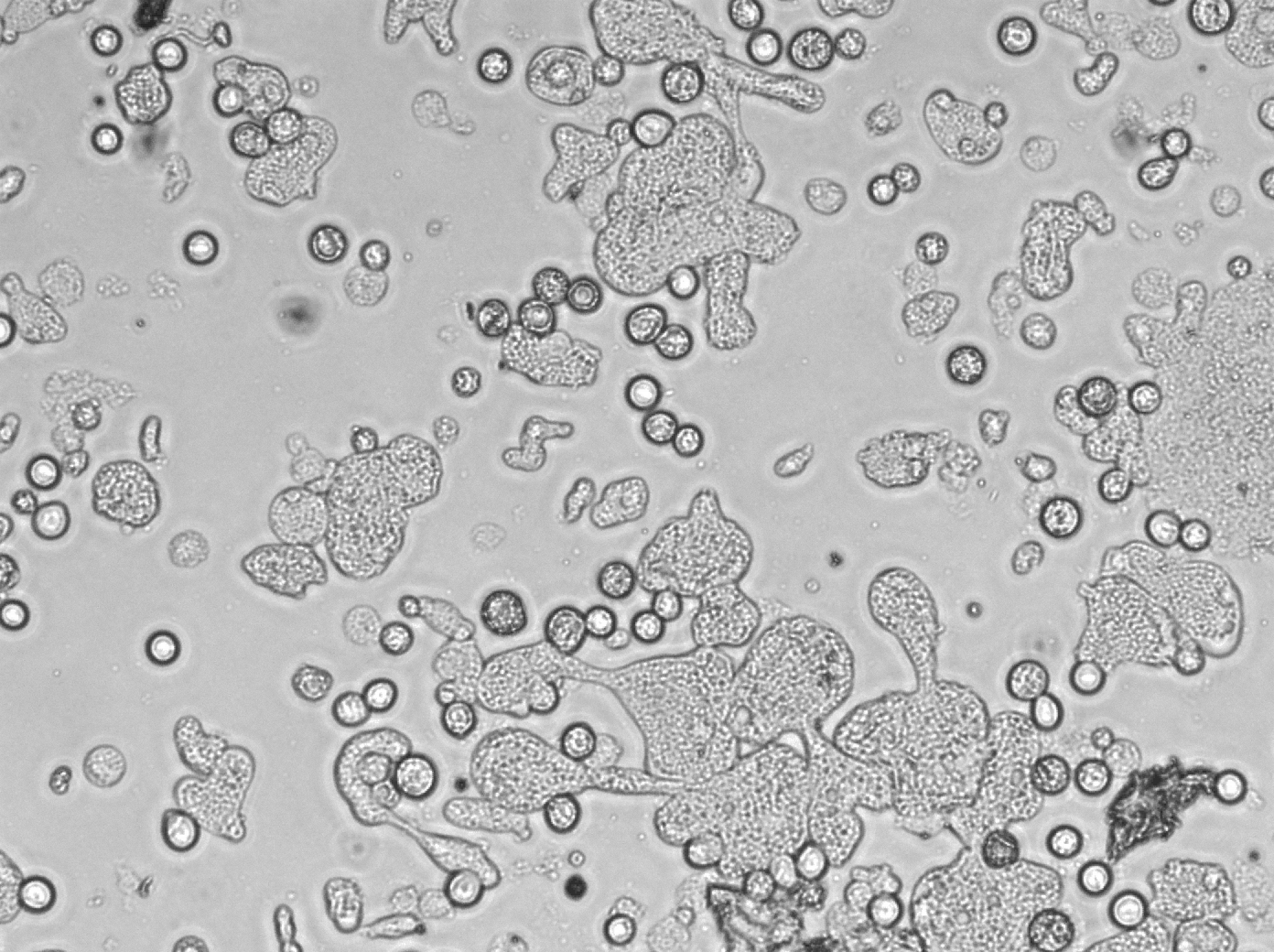


Movie S6. MGC reverts to trophozoites by continuous cytofission. Trophozoites and cysts can also be seen.


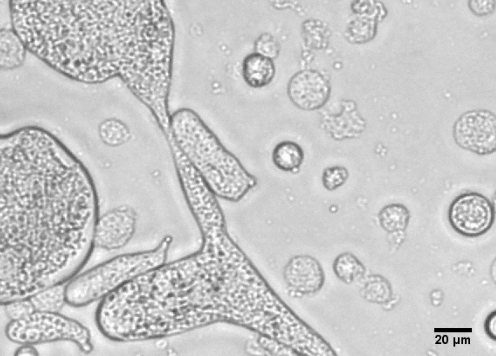


Movie S7. Assisted cytofission helped by midwife trophozoites.


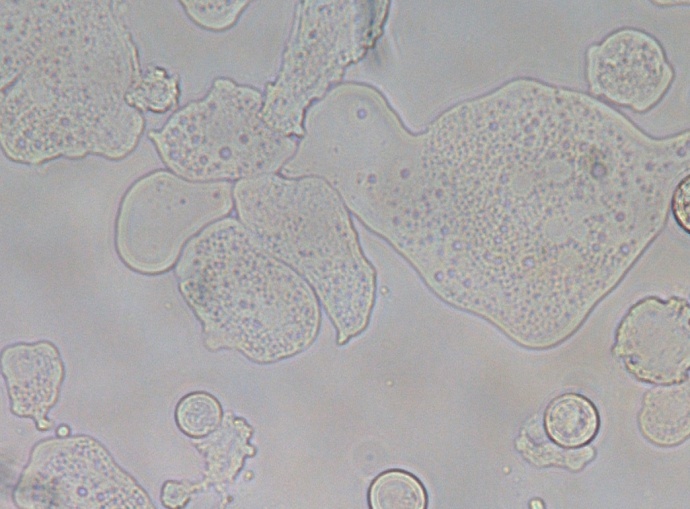


Movie S8. Assisted cytofission helped by midwife trophozoites.


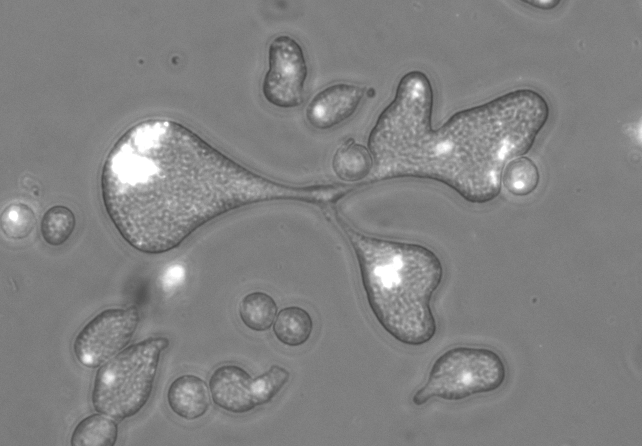


Movie S9. Random distribution of nuclei during cytofission.


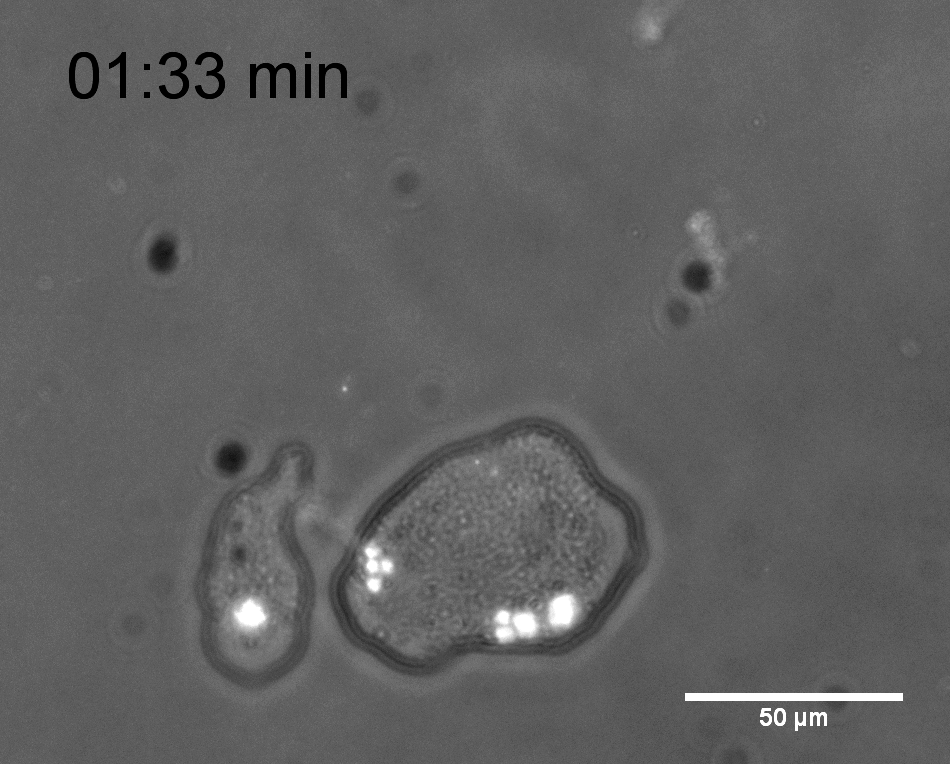


Movie S10. Early motile MGC with nuclei of different size taken from 24 hours encystation culture.


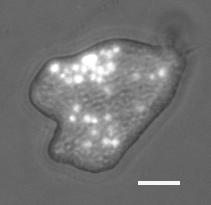


Movie S11. Motile MGC from 48 hours encystation culture showing dispersed nuclei.


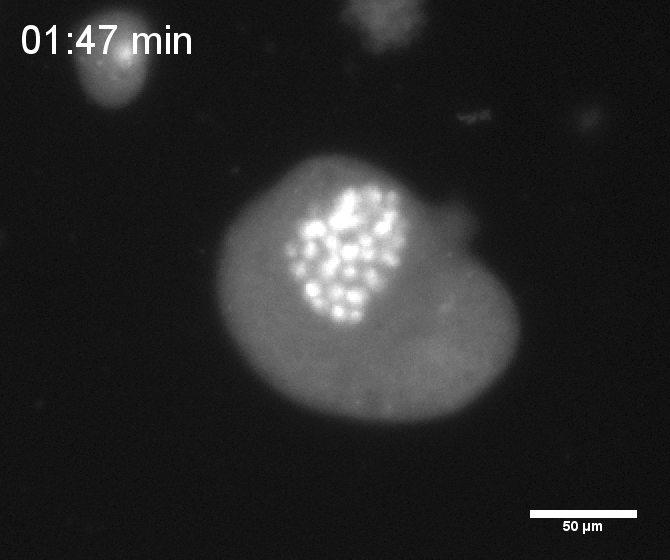


Movie S12. MGC from 72 hours encystation culture showing aggregated nuclei.
